# Infant and young child nutritional status and their caregivers’ feeding knowledge and hygiene practices in internally displaced person camps, Somalia

**DOI:** 10.1186/s40795-019-0325-4

**Published:** 2019-12-17

**Authors:** Mohamed Kalid, Fatumo Osman, Munshi Sulaiman, Fiona Dykes, Kerstin Erlandsson

**Affiliations:** 1 Save the Children International, Somalia and Dalarna University, School of Education, Health and Social Studies, Dalarna University, Falun, Sweden; 2 School of Education, Health and Social Studies, Dalarna University, Högskolegatan 2, 791 31 Falun, Sweden; 3 Research Evaluation, Monitoring, Learning and Monitoring (REALM) Save the Children International, Somalia Country Office, Nairobi, Kenya; 4 Fiona Dykes, Maternal and Infant Nutrition and Nurture Unit (MAINN), School of Community Health and Midwifery, University of Central Lancashire, Preston, Lancashire PR1 2HE UK

**Keywords:** Breastfeeding, Complementary foods, Hygiene, Education, Counseling, Internally displaced persons

## Abstract

**Background:**

In an attempt to design an educational programme targeting caregivers of children aged 6 to 59 months in internally displaced persons camps in Somalia, the objective of this study was twofold. First, to explore the nutritional situation of all children aged 6–59 months enrolled in a nutrition programme provided by Save the Children in 2017 in internally displaced persons camps. Second, to identify gaps in the caregivers’ hygiene and feeding practices.

**Methods:**

In a study of 1655 households, 1655 caregivers for 2370 children aged 6 to 59 months enrolled in a nutrition programme provided by Save the Children answered an adapted questionnaire on hygiene and feeding practices. At the same time, based on standard criteria in the questionnaire, naturalistic observations of caregivers’ hygiene practices were conducted. Every child in the study was measured with anthropometric Mid-Upper-Arm Circumference measurements for the classification of Moderate Acute Malnutrition, Severe Acute Malnutrition and Global Acute Malnutrition. Descriptive statistics were used for analysis.

**Results:**

1) There was Severe (12.1%) and Global Acute (19.9%) Malnutrition among children included in the nutrition programme, more frequently in the 6–24 month age group compared to the 25–59 month age group (*p* < 0.01). 2). The practices in the households were below what could generally be considered hygienic. 3) There was poor caregivers’ knowledge of breastfeeding benefits and complementary foods.

**Conclusion:**

Child malnutrition might derive from gaps in the caregiver’s knowledge, attitudes, and practices regarding hygiene and infant feeding. An awareness of these gaps can be helpful in designing future educational programmes that target caregivers, particularly in at-risk population groups.

## Background

Internally Displaced Persons (IDP) remain within their own country when they flee their homes due to natural disasters, violence and insecurity. In Africa, many countries play host to IDP populations in official camps and outside for shorter or longer period of time often with inadequate provision of housing and food supply in the camps. As a result of the uprooting, many IDP, particularly children, suffer from poor health and malnutrition. Studies have shown that IDP children suffer from high levels of illness and mortality [1–4]. For example, in a survey of IDP households in Nigeria, after liberation from Boko Haram, high levels of child mortality were reported that were above the emergency threshold. Researchers suggested that vaccinations and other preventative services could be used to treat the common childhood illnesses behind these figs [1].. After the political crisis in Uganda in East Africa in 2006, a cross-sectional study with 1080 IDP found three main physical health conditions: fever/malaria (48%), respiratory problems (45%), and depressive symptoms (67%) [2]. In Kenya, following post-election violence in 2007/2008, HIV infection and other chronic conditions were identified as significant causes of morbidity and mortality among their IDP population [3]. In a cross-sectional nutritional assessment survey from 2007 to 2010, with the aim of informing the better targeting of nutritional interventions, two of the predictors of malnutrition risk among children under the age of 5 in Somalia were the presence of infectious diseases and the likelihood of drought [5]. Somalia is a country with frequent humanitarian crises due to droughts and civil war. By 2018, more than 60% of Somali children had been uprooted because of the ongoing political unrest and repeated natural disasters. The deterioration of individual coping mechanisms and reduced communal resilience, which occurred as a result of eroded coping mechanisms, have affected how parents raise their children [4]. Internally displaced children and their caregivers, therefore, live under constrained conditions [6].

Located at the tip of the Horn of Africa, modern Somalia was formed out of former British and Italian colonies. In 1991 a bitter civil war commenced. The north-western part of the country broke away to form the autonomous region of Somaliland. A federal parliamentary system was established in Mogadishu, although fighting and political unrest has continued. Since the 1990s more than a million Somalis have become IDP. They share the same language and culture as their neighbours but lack homes, land, cattle or livelihood. Instead they are housed in IDP camps and outside of the official IDP camps [6]. Healthcare for IDP children has been of growing concern. Humanitarian agencies initially focused on providing food aid for IDPs, both inside and outside official IDP camps. As the conflict continued, and as long term stays in IDP camps became a reality, the focus has shifted towards healthcare provision. Alongside food supplies, humanitarian agencies began to provide Maternal and Child Health Clinics (MCH), Antenatal Care (ANC), vaccinations and health check-ups for children. The long-time civil war and recurring drought have not only contributed to children’s psychological suffering but also led to nutritional deficiencies and physical ill-health [7]. UNICEF estimated that 1.4 million children in Somalia were acutely malnourished by the end of 2017 [8]. Food insecurity has been assumed to be the explanation for these high levels of malnutrition [6]. In an attempt to respond to this humanitarian crisis, cash transfer programmes have been implemented. The role of an unconditional cash transfer, non-food item kit and free piped water to reduce malnutrition among children aged 6–59 months in the IDP camps has been investigated with a non-randomised cluster trial. While food security and wealth per household increased, no reduction of acute malnutrition in children was found [9, 10]. According to the World Bank, Somalia still has the highest under-five mortality rate in the world. In 2017, 127 per 1000 or one out of every eight children died before they were 5 years of age [11].

With this situation in mind a study was devised that would improve the effectiveness of life-saving actions in humanitarian emergencies; in particular, that would improve the design of educational programmes that target caregivers and their role in child health, nutrition and hygiene [12–14]. This study examined all caregivers with children 6 to 59 months taking part in a Save the Children nutrition programme in three IDP camps in three districts in Somalia. The research was conducted in August 2017, when a nationwide famine-like situation prompted Save the Children to scale up their humanitarian assistance. The objective was twofold. First to explore the nutritional situation of all children aged 6–59 months enrolled in a nutrition programme provided by Save the Children in IDP camps. Second to identify gaps in the caregivers’ hygiene and feeding practices. The study lent itself to a cluster sampling approach [15]. Gap identification of biological, social and cultural processes was carried out in cooperation with operational research and humanitarian agencies [12–14]. The underlying premise in this study was that formative research with key informants in different IDP camps could provide important insights to development agencies for improved educational programmes around child health and nutrition.

## Methods

### Design

All children aged 6–59 months living in the 1655 households and one caregiver per household enrolled in a nutrition programme provided by Save the Children in 2017 were included in the study. The nutrition programme was provided in three IDP camps in three different districts in Somalia. One caregiver from each of 1655 households answered a questionnaire on hygiene and feeding practices. At the same time, based on standard criteria in the questionnaire, naturalistic observations of caregivers’ hygiene practices were performed by the data collectors. Mid-Upper-Arm Circumference measurement was carried out for 2370 children [16]. Ethical approval was obtained from the Research and Ethics Review Committee of the Ministry of Health, South Central Somalia (D-nr: MoH& HS/DGO/0129/2017).

### Setting

The three selected IDP camps are located in the districts of Baidoa, Dharkenley and Dayniile. The camps in Dharkenley and Dayniile districts in the Benadir region were established in the twenty-first century. The selected IDP camp in the district of Baidoa in Bay region was established in the 1990s. Together they represent approximately 600,000 of the 1.1 million total Somali IDP population. The caregivers and the children in this study were living in the IDP camps in traditional houses, in so-called “aqals” built of straw or plastic with shared water supply and latrines. Goats, cattle, chicken, dogs roamed throughout the camps.

### Participants

As per the inclusion criteria, 1655 caregivers and 2370 children were included in the study. Caregivers and any of their children aged 6–59 months enrolled in a nutrition programme provided by Save the Children in 2017 were included. As part of the study’s ethical guidelines, Community Nutrition Volunteers’ (CNVs) observations which identified children with malnutrition and diseases such as pneumonia, malaria, measles or AIDS or with weights < 2500 g and the severely malnourished or ill were referred to health facilities for treatment and care [17]. The study used, ideally, vaccination cards to determine children’s ages. If there was no vaccination card or the caregiver did not recall their children’s dates of birth, a seasonal calendar was used as a tool to estimate age by mapping back through monthly changes in weather (rainfall or temperature) and agricultural activities.

### Questionnaire

The WHO Infant and Young Child Feeding Counselling (IYCF) questionnaire adopted by the research department of Save the Children, South Central Somalia was inspired by several other interrogative tools [18–20]. It was written first in English, then translated into Somali and back-translated into English as a built-in control to reduce bias [21]. The questions were pre-tested on 10 IDPs to ensure validity [15] before back-translation into English [21]. The feeding questions were, after pre-testing, adapted to the Somali context with regards to the specific foods available in South Central Somalia. The questions consisted of 98 closed and open yes/no or multiple-choice response alternatives in the following sections: demographic information on caregivers and households; household hunger; hygiene practices and observations; child feeding practices; and, infant and young child feeding (IYCF) knowledge and attitudes. For further details see a summary of the content of each section of the questionnaire in Appendix. Child anthropometry in terms of Mid-Upper Arm circumference [16] is included in the questionnaire as a subsection and described below.

#### Anthropometry

Malnutrition is divided into three measurements of the nutritional situation in a population; Moderate Acute Malnutrition (MAM), Severe Acute Malnutrition (SAM) and Global Acute Malnutrition (GAM). MAM and SAM are % of children below the anthropometric thresholds in a population and GAM is the sum of those. A GAM value of more than 10% indicates an emergency [22]. Humanitarian agencies use Mid-Upper-Arm Circumference as a anthropometric thresholds for admitting children with malnutrition to feeding programmes. The mid-point between the tip of the shoulder and the tip of the elbow is identified and then measured [23]. The cut-offs used by WHO to classify a child as MAM is a Mid-Upper-Arm Circumference between 110 mm and 125 mm, less than 110 mm is classified as SAM [16, 22]. A cut-off of point of 110 mm was used for SAM in this study. In some other studies where the Mid-Upper-Arm Circumference was measured the cut-offs has been increased from 110 mm to 115 mm to define SAM. This to ensure that children between are not missed out from management of malnutrition [22, 24, 25].

### Data collection

Twenty data collectors were recruited from rosters in the Mogadishu and Baidoa Save the Children offices. They were all professional data collectors with years of experience of gathering data for periodic monitoring and research projects. They received 2 days’ additional training before commencing their work for this study. This training was carried out at the end of July 2017 by the Save the Children research manager in the Save the Children office in Mogadishu. The training focused on administering the study questionnaire and how to make naturalistic observations. Intra-examiner reliability was established among the data collectors by a thorough discussion and testing of the questionnaire at the training sessions [15]. During the training, an online link to the questionnaire was distributed. The data collectors worked with Save the Children Community Nutrition Volunteers (CNV), who carried out the Mid-Upper-Arm Circumference measurements. The data collectors and CNVs gathered and recorded data 7 days a week throughout the month of August 2017.

The data collectors made one house visits to each of the 1655 households participating in the study. During the visit, data collectors provided verbal information about the twofold aim of the study, the design and assurances that participants could withdraw from the study without consequences at any time. The data collectors gained verbal consent from the guardians of the children. Informed consent was thus obtained from all 1655 caregivers and consent for the children involved before the data gathering began [17]. During the visit to the household, data collectors gave out appointment times and locations for the Mid-Upper Arm Circumference measurement and the completion of the questionnaire. It was at this visit that data collectors collected observational data on hygiene. Using naturalistic observation the real-life environment created by the household member’s behaviour was observed [15]. Household hygiene practices and environment were observed for an hour without providing advice to the caregivers. Corrective measures were suggested after the observation. Data collectors evaluated the presence of human faeces, garbage, and animal droppings (Appendix). By asking caregivers if their household experienced hunger often, rarely/sometimes or never during the past month/30 days, data collectors assessed hunger levels within the household [18]. The data collectors asked the caregivers to bring the child/children to the appointment at set time and place.

On the day of the appointment, to collect data at each IDP camp, data collectors and CNVs set up a collection centre in one of the ‘aqals’ in the IDP camp. CNVs set up their equipment for the anthropometric Mid-Upper-Arm measurements. At each appointment a CNV conducted the anthropometric Mid-Upper-Arm Circumference measurement/s of the child/children. The children turned up in a piece of cloths, or pants and a shirt. Children were examined in a standing position with their left/right arm hanging freely at their side. Because the arm measurement needed to be taken with a bare shoulder and arm, CNVs asked caregivers to remove any shirts with sleeves. For weak and young children, measurements were taken while in a recumbent position [16]. The procedure took approximately 10 min including information. Together with the caregiver and their child/children, the data collectors filled out the questionnaire. This process took approximately 30 to 45 min. The data collectors sent the completed questionnaires, including observations and anthropometric Mid-Upper-Arm Circumference measurement data, to the online server at Save the Children on a daily basis.

### Analysis

The information gained from the three districts (Baidoa, Dharkenley, and Dayniile) was entered into Stata© statistical software (StataCorp LLC™) [26], cleaned and analyzed using mean (m), standard deviation (SD) and percentage (%). The Chi-squared test (Pearson chi-squared), a non-parametric method for ordinal data, was used to analyze the differences between children aged 6–24 months and those aged 25–59 months for the classification of malnutrition and between the groups in each of the Baidoa, Dharkenley and Dayniile districts. Statistical significance was set at 0.05 [15].

## Results

There was Severe (12.1%) and Global Acute (19.9%) Malnutrition among children in these Somalian IDP camps in August 2017; this was more frequent in the age group 6–24 months than it was in the age group 25–59 months (*p* < 0.01). The frequency of malnourished children in the Dayniile district (*p* < 0.01) was higher in comparison to the Baidoa and Dharkenley districts (Table 1). Child marriage among the caregivers and illiteracy among household heads were frequently reported (Table 2). The use of ANCs and skilled birth attendants for deliveries in a health facility varied widely, from 89.2% in Baidoa, 9.5% in Dharkenley and 1.5% in Dayniile. For demographic data on caregivers (*n* = 1655) and households (*n* = 1655) see Table 2.

**Table 1:** Malnutrition based on Community Nutrition Volunteers’ measurements of Mid-Upper-Arm Circumference in children 6–59 months (*n* = 2370)

	^a^MAM	^b^SAM	^c^GAM
Malnutrition in number (n) per district, sex, and age	n(%)	n(%)	n(%)
Baidoa district (*n* = 1300)	103 (7.9)	73 (5.6)	176 (13.5)
Dharkenley district (*n* = 809)	71 (8.7)	27 (3.3)	98 (12.1)
Dayniile district (*n* = 261)	31 (11.9)	21 (8.0)	52 (19.9)
*p-value*	0.115	**0.005**	**0.006**
Male child (sex) (*n* = 1182)	113 (9.6)	62 (5.2)	175 (14.8)
Female child (sex) (*n* = 1188)	92 (7.7)	59 (5.0)	151 (12.7)
*p-value*	0.116	0.758	0.139
≤ 24 months of age (*n* = 824)	89 (10.8)	44 (5.3)	133 (16.1)
> 24 months of age (*n* = 1546)	116 (7.5)	77 (5.0)	193 (12.5)
*p-value*	**0.007**	0.705	**0.014**

^a^Moderate Acute Malnutrition (MAM)

^b^Severe Acute Malnutrition (SAM)

^c^Global Acute Malnutrition (GAM)

*p*-value <0.05 is marked with bold figures

**Table 2:** Demographic data on caregivers (n = 1655) and the situation in the households (*n* = 1655)

Demographic data on caregivers		Baidoa (*n* = 946)	Dharkenley (*n* = 573)	Dayniile (*n* = 136)
		n(%)	n(%)	n(%)
Age	15–24	238 (25.2)	236 (41.2)	30 (22.1)
	25–34	433 (45.8)	246 (42.9)	74 (54.4)
	35–44	230 (24.3)	80 (14.0)	29 (21.3)
	45 and above	45 (4.8)	11 (1.9)	3 (2.2)
Age at first birth	14–17	785 (83.0)	572 (99.8)	130 (95.6)
	18–34	149 (15.8)	1 (0.2)	6 (4.4)
	35–44	12 (1.3)	0	0
Age at first marriage	14–17	379 (40.1)	432 (75.4)	104 (76.5)
	18–34	560 (59.2)	141 (24.6)	32 (22.5)
	35–44	7 (0.7)	0	0
Age at first marriage	mean ± SD	18.9 ± 3.8	15.9 ± 1.9	16.3 ± 26
Demographic data on households (*n* = 1655)				
Head of household		n(%)	n(%)	n(%)
Head of household is female (n and %)		44 (4.7)	138 (24.1)	31 (22.8)
Head of family can read and write (n and %)		156 (16.5)	30 (5.2)	24 (17.6)
No direct income in household (n and %)		199 (21.0)	218 (38.0)	30 (22.1)
Household hunger		n(%)	n(%)	n(%)
Often (n and %)		427 (45.1)	26 (4.5)	5 (3.7)
Rarely/Sometimes (n and %)		519 (54.9)	547 (95.5)	131 (96.3)
Never (n and %)		0	0	0

Hygiene (*n* = 1655) was below what can be considered a result of hygiene practices (Table 3).

**Table 3:** Caregivers’ statements and data collectors’ naturalistic observations on hygiene practices (*n* = 1665)

Hygiene practices		Baidoa (*n* = 946)	Dharkenley (*n* = 573)	Dayniile (*n* = 136)
Caregivers’ statements		n(%)	n(%)	n(%)
No treatment for drinking water		203 (21.5)	151 (26.4)	74 (54.4)
Drinking water treated with boiling		231 (24.4)	299 (52.2)	58 (42.6)
Drinking water treated with tablets		512 (54.1)	123 (21.5)	4 (2.9)
Adult family members wash hands with soap before a meal	Never	148 (15.6)	117 (20.4)	22 (16.2)
	Sometimes	460 (48.6)	416 (72.6)	113 (83.1)
	Always	338 (35.7)	40 (7.0)	1 (0.7)
Children wash hands with soap before a meal	Never	225 (23.8)	103 (18.0)	22 (16.2)
	Sometimes	586 (61.9)	366 (63.9)	113 (83.1)
	Always	135 (14.3)	104 (18.2)	1 (0.7)
Adult family members wash hands with soap after defecation	Never	192 (20.3)	61 (10.6)	12 (8.8)
	Sometimes	476 (50.3)	379 (66.1)	116 (85.3)
	Always	278 (29.4)	133 (23.2)	8 (5.9)
Children wash hands with soap after defecation	Never	232 (24.5)	67 (11.7)	33 (24.3)
	Sometimes	600 (63.4)	384 (67.0)	99 (72.8)
	Always	114 (12.1)	122 (21.3)	4 (2.9)
Naturalistic observations		n(%)	n(%)	n(%)
Caregiver’s hands are clean		381 (40.3)	258 (45.0)	1 (0.7)
Caregiver’s clothes are clean		349 (36.9)	292 (51.0)	23 (16.9)
Interior of house looks swept		501 (53.0)	268 (45.9)	85 (62.5)
Drinking container is covered		602 (63.0)	321 (56.0)	50 (36.8)
Human faeces observed around the house		434 (45.9)	89 (15.5)	47 (34.6)
Animal droppings observed around the house		485 (51.3)	142 (24.8)	68 (50.0)
Household waste observed around the house		488 (51.6)	171 (29.8)	92 (67.6)

Caregivers’ statements on child feeding practices (*n* = 1655) indicate the presence of unhealthy practices, such as giving animal milk to a child under 6 months in Baidoa *n* = 459 (48.5%), in Dharkenley *n* = 299 (52.2%) and in Dayniile *n* = 120 (88.2%), using water to quench the thirst of newborn infants Baidoa *n* = 583 (61.6%), Dharkenley *n* = 267 (46.6%), Dayniile *n* = 132 (97.1%), perceiving colostrum to be harmful to the health of a child Baidoa *n* = 485 (51.3%), Dharkenley *n* = 360 (62.8%), Dayniile *n* = 46 (33.8%) and giving fewer liquids than normal to a child with diarrhoea Baidoa *n* = 211 (22.3%), Dharkenley 161 (28.1%), Dayniile *n* = 77 (56.6%) (Table 4). Table 5 presents the knowledge and attitudes of caregivers regarding infant and young child feeding. Gaps identified were the unhealthy practices of sugar or glucose water to children under 6 months. Half to two-thirds of the caregivers stated that a newborn baby should be put on the breast to suckle within an hour after birth and that breastfeeding should be continued throughout the entire complementary foods period until the child reached the age of two. Most caregivers reported that the introduction of solid and semi-solid food should be introduced to children after 6 months of age. Food that could be introduced to a child during the complementary food period included thin porridge, rice, vegetables, fruits and fruit juice, egg yolk or whole eggs, meat, fish and poultry.

**Table 4:** Caregivers’ statements on child feeding practices (*n* = 1665)

	Baidoa (*n* = 946)	Dharkenley (*n* = 573)	Dayniile (*n* = 136)
Caregivers agreed to the following statements:	n(%)	n(%)	n(%)
Animal milk (goat and camel) should be given to children < 5 months	459 (48.5)	299 (52.2)	120 (88.2)
A 3 day-old baby needs water to quench its thirst	583 (61.6)	267 (46.6)	132 (97.1)
Breastfeeding increases mother-child bonding	714 (75.5)	424 (74.0)	125 (91.9)
First yellow breast milk (danbar, colostrum) is harmful to children’s health	485 (51.3)	360 (62.8)	46 (33.8)
A small child with diarrhoea should be given fewer liquids than normal	211 (22.3)	161 (28.1)	77 (56.6)

**Table 5:** Caregivers’ knowledge and attitudes regarding infant and young child feeding (*n* = 1665)

	Baidoa (*n* = 946)	Dharkenley (*n* = 573)	Dayniile (*n* = 136)
The caregivers agreed with the following statements:			
*A newborn baby should be put on the breast to suckle:*	n(%)	n(%)	n(%)
within an hour after birth	705 (74.5)	327 (57.1)	12 (8.8)
between 1 and 2 h after birth	173 (18.3)	224 (39.1)	118 (86.8)
more than 2 h after birth	57 (6.0)	22 (3.8)	6 (4.4)
*An infant should be fed on the mother’s breast milk without other solid or non-solid food items (even without water) for:*	n(%)	n(%)	n(%)
up to 3 months	232 (24.5)	146 (25.5)	17 (12.5)
up to 6 months	590 (62.4)	273 (47.6)	115 (84.6)
*The following foods could be introduced to an infant in addition to breast milk*	n(%)	n(%)	n(%)
Sugar water or glucose water			
under 6 months	178 (18.8)	289 (50.4)	115 (84.6)
between 6–8 months	401 (42.3)	217 (37.9)	19 (14.0)
above 8 months	314 (33.2)	65 (11.3)	0
Thin porridge			
under the age of 6 months	70 (7.4)	69 (12.0)	18 (13.2)
between the age of 6–8 months	221 (23.4)	279 (48.7)	102 (75.0)
above the age of 8 months	583 (61.6)	204 (35.6)	11 (8.1)
Rice			
under the age of 6 months	58 (6.1)	27 (4.7)	23 (16.9)
between the age of 6–8 months	181 (19.1)	243 (42.4)	36 (25.5)
above the age of 8 months	634 (68.0)	281 (49.0)	76 (55.9)
Vegetables			
under the age of 6 months	64 (6.8)	24 (4.2)	20 (14.7)
between the age of 6–8 months	189 (20.0)	176 (30.7)	31 (22.8)
above the age of 8 months	604 (63.8)	349 (60.9)	82 (60.3)
Fruits and fruit juice			
under the age of 6 months	66 (7.0)	22 (3.8)	24 (17.6)
between the age of 6–8 months	195 (20.6)	217 (37.8)	62 (45.6)
above the age of 8 months	574 (60.9)	313 (54.6)	48 (35.3)
Eggs (yolk and whole)			
under the age of 6 months	70 (7.4)	55 (9.6)	16 (11.8)
between the age of 6–8 months	190 (20)	209 (36.5)	78 (57.4)
above the age of 8 months	506 (53.5)	281 (49)	39 (28.7)
Meat/fish/poultry flesh			
under the age of 6 months	69 (7.3)	43 (7.5)	11 (8)
between the age of 6–8 months	164 (17.3)	162 (28.3)	25 (18.4)
above the age of 8 months	509 (53.8)	337 (58.8)	96 (70.6)
*A baby between 6–8 months should be fed in a day:*	n(%)	n(%)	n(%)
once or less	259 (27.4)	52 (9)	8 (5.9)
twice or more	644 (68)	508 (88.7)	127 (93.4)
*An infant should continue to breastfeed in addition to other complementary solid and non-solid food items up to the age of:*	n(%)	n(%)	n(%)
1 year	386 (40.8)	129 (22.51)	33 (24.26)
2 years	509 (53.8)	432 (75.4)	102 (75.0)

## Discussion

The nutrition situation in Dayniile was critical (GAM 19.9%), whereas in Dharkenley and Baidoa the situation was serious (GAM 12.1 and 13.5% respectively). With the Somali national GAM level sitting at 17.4%, the situation in two of the three districts included in this study was actually less serious for children compared to the national average [6, 27, 28]. The MAM in Dayniile (8.0%), Baidoa (5.6%) and Dharkenley (3.3%) can be compared with the national MAM figure of 3.2% indicating there was more MAM for the children included in this study than the national data indicated [6, 27, 28]. With figures like this it goes without saying that SAM is endemic for Somali children on a national level. In one systematic review, the risk of malnutrition in Somalia was shown to be largely predictable. A key predictor of malnutrition was in a previous study shown to be the presence of disease, such as HIV and other chronic conditions. It was concluded that healthcare and support efforts had to include infected IDPs located outside of IDP camps as well as those within if malnutrition was to be avoided [4]. Another key predictor of malnutrition was the lack of close monitoring of drought forecasts. The study showed that closer monitoring of climatic trends could provide valuable information for nutritional intervention planning [5]. In 2017, when our study was carried out, a nationwide famine was making assistance necessary to IDPs both in the camps and outside of them. Humanitarian agencies were scaling up their efforts to reach the nearly 1.2 million children under five who were acutely malnourished [8]. The Food and Agriculture Organization of the United Nations [29], continues to report widespread shortages of water across Somalia, nearly 1.7 million people living in emergency situations and a further 72.869 people being displaced. Researchers now agree that gaps in local caring practices should be identified as a baseline prior to scaling up interventions [13, 14, 30].

The use of ANCs and skilled birth attendants for deliveries in a health facility varied widely, from 89.2% in Baidoa, 9.5% in Dharkenley and 1.5% in Dayniile. This is a striking difference that can influence nutrition knowledge and hygiene practices. The IDP camp in Baidoa was since decades established compared to the IDP camps in Dharkenley and Dayniile established in the twenty-first century. The time from establishment might influence women’s ANC visits and facility-based births. The contact with health care providers at the health facility can influence women’s nutrition knowledge and hygiene practices. This is interesting because the households in Baidoa reported high hunger levels but they had fairly decent hygiene practices. And it looks like households in the Dayniile had poor hygiene practices though they reported low hunger levels. The acceptability and accessibility of health professionals in the health facilities might differ between the camps due to the time from establishment. The gaps in the hygiene and feeding practices identified in this study can be used to build the capacity of local caregivers, through counselling or education, in areas such as hygiene, breastfeeding, and complementary feeding practices. Advice from health professionals at MCH clinics can help to promote the benefits of colostrum, early initiation of breastfeeding after birth and exclusive breastfeeding for 6 months continued to 24 months with complementary feeding practices. This is consistent with previous research and recommendations on counselling, education and programme implementation [31–33] in various settings.

According to the Sustainable Development Goals (SDGs) [34, 35] the potential for a child to thrive starts with pregnancy and is particularly important in the first months and years when children are most vulnerable to sickness and malnutrition. Consequently, a cash-based intervention and the risk of acute malnutrition in children aged 6–59 months living in IDP camps in Mogadishu, Somalia was undertaken in 2016. The conclusion was that cash-based interventions improved wealth and food security but did not reduce acute malnutrition in children. The authors suggested the addition of specific nutritious foods and more communication around social and behavioural change would be more effective [10].

Sustainable humanitarian assistance in terms of counselling or education in care practices may enhance caregivers’ resilience in the long-term [31] when combined with other emergency efforts from time to time [4, 5]. The distress caused by long term insecurity, lack of income and drought and the effect of recurrent emergencies on the emotional well-being and mental health of caregivers and children was not investigated in this study although it needs further attention. In an upcoming study the correlations between malnutrition and care practices considering water, sanitation and hygiene will be more closely examined. The results, it is hoped, will further contribute to the understanding of effective interventions in malnutrition.

This study showed that children aged 24 months were most vulnerable to malnutrition. This may be a result of the occasional hunger situation in participant households and the presence of unhealthy practices regarding hygiene and feeding. After the acute phase of humanitarian assistance, a more sustainable phase of humanitarian assistance and the reintegration of IDPs has been emphasized [4, 27, 28]. Sustainable humanitarian assistance in the form of clean water supplies and sanitation modalities is one fundamental strategy for health; another is education. Hygiene and cleanliness are well-known keys to the health and growth of children [4, 30]. Universal, affordable and sustainable access to water, sanitation, and hygiene (WASH) is a public health effort focusing on SDG 6 [30, 35]. It includes behaviour change communication for caregivers with limited education including depression and stressful situations, it will help development agencies to design the best programmes for caregivers in the future [30].

## Limitations

The main limitation of the data was regarding construct validity [15] and the lack of statistical correlations across the data. Alpha-reliability was not measured. Instead, we chose to rely on conclusion validity among the authors of this study, discussing and reflecting on similarities and differences between and across the data. The decision was taken to present the data from the three districts separately to provide readers with a more nuanced picture of IDPs across Somalia. The results from Baidoa could reflect its long history of humanitarian aid and suggest that caregivers there knew more about child health and nutrition than in the other two districts. It could be seen as a strength of our study that it was possible to compare our results with country data [15].

In our experience, social-acceptability bias [15] may be a problem with some of the questions in the questionnaire. For example, mothers answered that they always washed their hands before preparing food or before a meal, but when naturalistic observations were conducted, based on standard criteria in the questionnaire, this washing did not seem to have taken place. The fact that reported household hunger levels did not fully correspond with child anthropometry Mid-Upper-Arm Circumference (*n* = 2370) suggests that caregivers may have underreported hunger levels by disproportionately choosing the ‘rarely/sometimes’ option. The utilization of the Mid-Upper-Arm Circumference measurement [16], despite its limitations, could be seen as strengthening reliability when carried out by well-trained CNVs and recorded by experienced data collectors.

Intra-examiner reliability of the questionnaire was established through consensus validity among the data collectors at the training event organised before the commencement of data collection and pilot testing. Regarding generalization, there are several weaknesses in this study [15]. It is important for further studies to use random sampling to avoid biased results; to present the effect of birth order on malnutrition when more than one child is included from a household and weight for height in addition to the Mid-Upper-Arm measurements. Concurrently with this study, the USAID [36] and UN Refugee Agency (UNHCR) described similar results [37]. Hence, the recommendations from this study, with caution, can be applied not only to Somali internally displaced children and their caregivers but can be generalized to similar IDP settings elsewhere in the world.

## Conclusion

This study provides information on risk factors for malnutrition of children 6–59 months from three districts of Somalia with frequent humanitarian crises. The risk factors for malnutrition in this study derive from gaps in the caregiver’s knowledge, attitudes and practices regarding hygiene and feeding. The data can be helpful in documenting child malnutrition levels in Somalia and prioritizing the actions needed to combat these. The information on gaps can be helpful in designing future educational programmes for caregivers in at-risk population groups. The results can be presented to local authorities in the planning phase of humanitarian aid interventions.

## Data Availability

All of the data and materials related to this study are available upon request from Save the Children, Somalia Country office. The data is stored on the Save the Children server.

## Appendix

### Content of the sections in the Infant and Young Child Feeding (IYCF) questionnaire

#### Demographic information on caregivers and households

##### Household hunger

In the *Hunger in the household* section (Table 2) all available response alternatives are presented for the three questions, with the response alternatives: *never*, *rarely/sometimes* (1–10 times per 30 days) and *often* (> 10 times per 30 days). The questions were: “In the past 4 weeks/30 days was there no food to eat of any kind in your house because of lack of resources to get food? In the past 4 weeks/30 days did you or any household member go to sleep at night hungry because there was not enough food? In the past 4 weeks/30 days did you or any household member go a whole day and night without eating anything at all because there was not enough food?”

##### Hygiene practices and observations

*Hygiene practices and observations* of hygiene practices included questions to caregivers and additional naturalistic observations made by the data collectors. For example, the question “How do you treat drinking water?” was asked with the response alternatives being: 0 = not treated, 1 = boiling, 2 = tablets. This question is presented in Table 3 alongside total results for hygienic practices, where 1 = boiling + 2 = tablets. Questions about adult and children family members handwashing with soap before a meal and after defecation included the response alternatives 0 = never, 1 = sometimes and 2 = always. Data collectors observed the caregivers’ clothes with the alternatives 1 = dirty, 2 = dusty, or 3 = clean. The clean alternative is presented.

The questions: “Does the interior of the house look like it needs to be swept?”, “Is drinking containers covered?”, “Can human faeces, animal droppings, garbage be observed around the house?” came with the closed response alternative Yes/No.

##### Child feeding practices

*Child feeding practices* with caregivers’ statements the use of colostrum, animal milk or water, infant bonding and the treatment of diarrhoea had the response alternatives of 1 = agree, 2 = disagree and 3 = not sure. This will be reported on with the agree alternatives in Table 4.

##### Infant and Young Child Feeding (IYCF) knowledge and attitudes

In the section *IYCF knowledge and attitudes* (Table 5) caregivers’ knowledge and attitudes are presented in response to the following questions: “How long after birth a baby should be put to the breast to suckle and for how long an infant should be fed on the mother’s breast milk without other solid or non-solid food items (even without water)?” and “At what age should the following foods be introduced to an infant in addition to breastmilk?” A set of food alternatives are presented in Table 5 with response alternatives before 6 months, between 6 and 8 months and after 8 months of age. “In a typical day, how many times should a baby aged between 6-8 months be fed?” was asked with response alternatives 1 = once or less, 2 = twice or more, and “How long should an infant continue breastfeeding in addition to other complementary solid and non-solid food items?” was asked with response alternatives 1 = up to one year, 2 = up to 2 years.

#### Anthropometry

Mid-Upper Arm Circumference MAM >110 mm - <125 SAM <110 mm with/without bilateral pitting odema.

## Abbreviations

CNV: Community Nutrition Volunteers
GAM: Global Acute Malnutrition
IDP: Internally Displaced Person
IYCF: Infant and Young Child Feeding
MAM: Moderate Acute Malnutrition
SAM: Severe Acute Malnutrition
